# Phytochemical profiling and antidiarrheal activity of *Sophora japonica* L. fruit extract

**DOI:** 10.1007/s44446-026-00081-3

**Published:** 2026-04-16

**Authors:** Fathia S. Elshaarawy, Shimaa K. Mohamed, Mohamed I. S. Abdelhady, Nermine M. Mohammed

**Affiliations:** 1 Department of Pharmacognosy, Faculty of Pharmacy, Helwan University, Ain Helwan, Cairo, 11795 Egypt; 2 Department of Pharmacology and Toxicology, Faculty of Pharmacy, Helwan University, Ein Helwan, Cairo, 11795 Egypt

**Keywords:** *S. japonica*, antioxidant and antidiarrheal activities

## Abstract

One of the biggest causes of disease and death worldwide is still diarrhea, particularly affecting children in developing nations. Genus *Sophora* (Fabaceae) comprises over fifty species known for their diverse bioactive secondary metabolites and notable pharmacological benefits. This investigation aimed to isolate and identify the polyphenol components in an 80% aqueous methanolic extract (AME) of *Sophora japonica* fruits and to assess its antioxidant and antidiarrheal properties. A castor oil-induced diarrhea model in rats was used to assess antidiarrheal efficacy, with animals divided into five groups: three groups treated with AME at 250, 500, and 1000 mg/kg; a control group; and a reference group treated with loperamide (5 mg/kg). Seven compounds were isolated, including **compound 1** (cinnamic acid), **compound 2** (gallic acid), **compound 4** (rutin), **compound 5** (genistein), **compound 6** (isoquercitrin), **compound 7** (quercetin), and **compound 3,** a new compound identified as (4′-*O*-methylgenistein-6′′′-acetyl-sophoroside). The extract decreased both total and wet fecal production in a dose-dependent manner. It markedly delayed the onset of diarrhea, demonstrating an antidiarrheal index (ADI) of 71.75, comparable to that of the conventional medication at 1000 mg/kg. These results highlight *S. japonica* fruit extract as a promising natural source for the development of antidiarrheal agents.

**Supplementary Information:**

The online version contains supplementary material available at 10.1007/s44446-026-00081-3.

## Introduction

Diarrhea is still a serious global public health concern, particularly in low- and middle-income nations where it greatly increases childhood morbidity and mortality. It is the second most frequent cause of mortality for children under five, according to the World Health Organization. The condition is characterized by frequent passage of watery or loose stools, often leading to dehydration and electrolyte imbalance (Kumar et al. 2023). The underlying causes may include infections by bacteria, viruses, or parasites, intestinal inflammation, adverse drug reactions, and dietary factors (Dos Santos Negreiros et al. 2019; Kumar et al. 2023). Diarrheal episodes are generally classified by mechanism or duration. Mechanistically, they can be osmotic, secretory, or motility-related. Osmotic diarrhea results when unabsorbed solutes retain water in the intestinal lumen, whereas secretory diarrhea arises from excessive ion and water secretion due to enterocyte dysfunction or toxin stimulation (Murugan et al. 2020). Clinically, diarrhea may be acute (lasting less than 14 days), persistent (lasting 2 weeks or more), or chronic (lasting more than a month) (Dos Santos Negreiros et al. 2019). Despite their effectiveness, conventional antidiarrheal medications can have unfavorable side effects. Moreover, the emergence of resistance underscores the need for safer, plant-based alternatives. Medicinal plants have long served as therapeutic sources, with approximately 25% of modern pharmaceuticals derived from higher plants. It is estimated that up to 80% of the global population depends on herbal medicine for primary healthcare (Brinsi et al. 2024). The genus *Sophora* (Fabaceae) includes more than fifty species and several varieties distributed mainly across Asia and parts of Africa. Members of this genus are rich in bioactive secondary metabolites, including coumarins, alkaloids, triterpenoid saponins, flavonoids, isoflavonoids, and other phenolic compounds. Extracts and isolated constituents from *Sophora* species exhibit diverse pharmacological properties, including antioxidant, antibacterial, anti-inflammatory, anticancer, antidiabetic, and antidiarrheal effects (Sweelam et al. 2018; Boozari et al. 2019; Elberry et al. 2020). Traditionally, species such as S. flavescens, S. tonkinensis, and S. alopecuroides have been used in Asian medicine to treat acute dysentery, gastrointestinal disturbances, and inflammatory conditions (Abd-Alla et al. 2021). Given this pharmacological potential, the present study sought to isolate polyphenolic compounds from *S. japonica* fruit extract and to evaluate its antioxidant and antidiarrheal efficacy using in vivo models.

## Materials and methods

### General experimental material

#### Chemicals and reference standards

Authentic standards for sugars and flavonoids were obtained from the Pharmacognosy Department, Faculty of Pharmacy, Helwan University, Egypt. Authentic standards for gallic and cinnamic acids were purchased from Sigma-Aldrich (Merck). Reagents used included Polyamide S6 (Fluka Chemie AG, Switzerland), Microcrystalline cellulose (E. Merck, Germany), and Sephadex LH-20 (Pharmacia, Sweden). For chromatographic analyses, silica gel 60 F254 and cellulose precoated sheets (E. Merck) were used for thin-layer chromatography (TLC). In contrast, Whatman No. 1 paper was used for paper chromatography (Whatman Ltd., Maidstone, Kent, England). All of the following chemicals were obtained from commercial sources. They were used without further purification: diphenylborinic acid ethanolamine complex (for flavonoids), aluminum chloride (1% in ethanol), ferric chloride (1%), aniline hydrogen phthalate, nitrous acid spray (for ellagitannins), and potassium iodate (for gallotannins). Sodium methoxide, aluminum chloride, boric acid, HCl, and sodium acetate were among the reagents used for UV spectrum analysis, following established procedures (Markham 1982; Hiermann 1983).

#### Plant material

*S. japonica* fruits were gathered from planted trees at Cairo University’s Faculty of Pharmacy’s Medicinal Plant Station. Associate Professor Dr. Sherif El-Khanagry of the Agriculture Museum in El-Dokki, Cairo, Egypt, verified the identification. The Pharmacognosy Department received a voucher specimen (No. 9*Sja*16/2025).

#### Solvent systems

Three solvent systems were utilized for (TLC) chromatography:

- **S1:** n-Butanol–Acetic acid–Water (4:1:5, upper phase)
- **S2:** Acetic acid–Water (15:85, v/v)
- **S3:** n-Butanol–Isopropyl alcohol–Water (4:1:5, upper phase)

#### Drugs

The in vivo antidiarrheal tests used castor oil, 10% charcoal suspended in 5% gum acacia, and loperamide (5 mg/kg) as the antidiarrheal standard. Charcoal and castor oil were purchased from a general market, and loperamide was acquired from Sigma-Aldrich in Missouri, USA.

#### Equipment

A rotary evaporator (Buchi AG, Switzerland) was used for solvent removal. Chromatographic separations were performed in glass columns (120 × 3.5 cm). TLC and PC tanks of appropriate sizes were used for running TLC. UV light was used for visualization. Nuclear Magnetic Resonance (NMR) spectra were obtained using TMS as an internal standard and DMSO-d6 as a solvent on a Bruker 400 MHz spectrometer for ^1^H and a 100.4 MHz spectrometer for ^13^C.

### Determination of total phenolic contents

Gallic acid was used as a calibration standard in the Folin-Ciocalteu method (Kumar et al. 2008) to measure the total phenolic content (TPC) in the 80% aqueous methanol extract (AME) of *S. japonica* fruits. In short, 500 μL Folin-Ciocalteu reagent, 1.5 mL sodium carbonate (20%), and 100 μL extract (100 μg/mL) were combined, diluted to 10 mL with distilled water, and incubated for two hours. The absorbance was measured at 765 nm, and the following formula was used to represent the results as mg gallic acid equivalents (GAE) per g:

$$T=\frac{C\times V}{M}$$

where *C* is the gallic acid concentration (mg/mL), *V* is the extract volume (mL), and *M* is the extract weight (g).

### Determination of total flavonoid contents

Quercetin was used as a standard in the aluminum chloride colorimetric method (Kumaran and Karunakaran 2006) to measure the flavonoid concentration. Lithium trichloride reagent was combined with an aliquot (100 μL) of the methanolic extract (100 mg/mL) and diluted to 500 μL with methanol. Absorbance was measured at 415 nm against a blank after 40 min. Quercetin equivalents (QE) in milligrams per gram were used to express the results.

### Extraction and isolation of polyphenolic constituents from *S. japonica* fruits

**Fig. 1:**
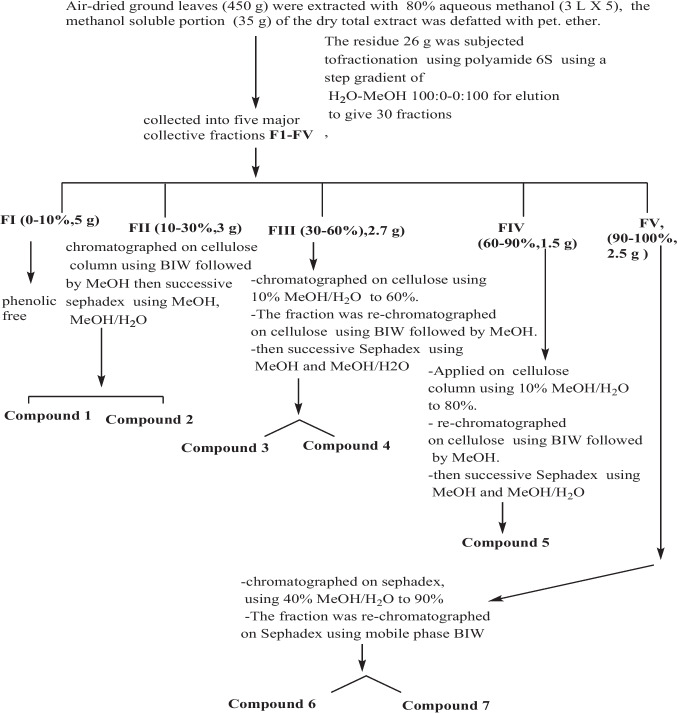
Scheme for isolation of polyphenolic constituents from *S. japonica* fruit AME

Air-dried ground fruits (450 g) were extracted with hot 80% aqueous methanol (3 L × 5) by water bath (60˚C). The methanol-soluble portion of the dry total extract (35 g) was defatted with petroleum ether. ether (60˚C) under reflux (1 L × 5). The residue was subjected to fractionation using polyamide 6S (Riedel De Haen AG, Seelze, Hannover, Germany) column (300 g, 110 X 7 cm) using a step gradient of H_2_O-MeOH 100:0–0:100 for elution to give 30 fractions of the first residue which were collected and monitored by comparative PC (systems S_1_ & S_2_) and UV-light into five major collective fractions as shown in Fig. 1.

Fraction I (FI 0–10% MeOH/H_2_O, 5 g) was phenolic-free. Fraction II (FII 10–30%, 3 g) was chromatographed on cellulose column using BIW (4:1:5, upper phase) followed by MeOH then successive sephadex LH-20 column for separation using mobile phase MeOH and (MeOH/H_2_O 20–30%) to afford **compound 1** (20 mg) and **2** (25 mg), R_f_ values were (0.6, 0.3 in S3 respectively. Fraction III (FIII 30–60%, 2.7 g) was chromatographed on a cellulose column using 10% MeOH/H_2_O with decreasing polarity to 60% MeOH/H_2_O. The fraction was re-chromatographed on another cellulose column using BIW followed by MeOH. Successive Sephadex LH-20 column for separation using mobile phase MeOH and (MeOH/H_2_O 30:70%) to afford **compound 3** (15 mg) and **4** (20 mg), R_f_ values were (0.35, 0.44 in S3, respectively. Fraction IV (FIV 60–90%, 1.5 g) was chromatographed on a cellulose column using 10% MeOH/H_2_O with decreasing polarity to 80%. The fraction was re-chromatographed on a cellulose column using BIW followed by MeOH, then a successive Sephadex LH-20 column using MeOH and (MeOH/H_2_O 50:50) to afford **compound 5** (18 mg), R_f_ values were (0.6 in S3). Fraction V (FV 90–100%, 2.5 g) was chromatographed on a Sephadex column, and the column was eluted using 40% MeOH/H_2_O with decreasing polarity to 90% MeOH/H_2_O. The fraction was re-chromatographed on a Sephadex LH-20 column for separation using mobile phase BIW to afford **compound 6** (20 mg) and **7** (22 mg), R_f_ values were (0.72, 0.96 in S3, respectively.

### Antioxidant activity

The DPPH technique was used to assess the capacity to scavenge free radicals (Mensor et al. 2001). Gallic acid (10–100 µg/mL) was used as a standard, and extracts were tested at 100–1000 µg/mL in methanol. Once 1 mL of 0.2 mM DPPH and 1 mL of the extract solution were combined, the mixture was allowed to sit at room temperature in the dark for 30 min. At 517 nm, absorbance was measured. The inhibition % was computed as follows:

$$I\mathrm{\%}=\frac{({A}_{0}-{A}_{1})}{{A}_{0}}\times 100$$

where the absorbance values of the control and sample are denoted by A₀ and A₁, respectively.

### In vivo procedure of antidiarrheal assessment

#### Animals

We obtained 42 Swiss albino mice (20 ± 5 g) for an acute toxicity test and 30 female Sprague–Dawley rats (180 ± 10 g) for assessing antidiarrheal effects from the VACSERA domestication unit (Helwan, Cairo, Egypt). For 1 week prior to the study, we housed the rats and mice in standard cages (3 rats/mice per cage) to ensure proper adaptation. They were preserved on a 12-h light/dark cycle at 23 ± 2 °C. The rats were given unlimited access to water and regular food. The Helwan University Faculty of Pharmacy’s Institutional Animal Care and Use Committee (IACUC No: 26A2025) reviewed and approved all research processes.

#### Evaluation of acute oral toxicity

In short, two mouse groups were created for the experiment: the assessed group and the control group. The assessed group was given oral doses of the AME (50 mg/kg, 200 mg/kg, 500 mg/kg, 1 g/kg, 2.5 g/kg, and 5 g/kg). In contrast, the control group has received ordinary saline. The mice’s overall behavior and the percentage of deaths were recorded for 2 days. Mady et al. (2022) defined a safe extract as one that does not affect mice at doses up to 5 g/kg. Therefore, the investigational dosages utilized in this investigation were 250, 500, and 1000 mg/kg.

#### Stimulation of diarrhea in rats by castor oil administration

Five groups (*n* = 6) of fasted rats (18 h) were aimlessly assigned: Three test groups were given oral doses of AME of *S. Japonica* fruit at 250, 500, and 1000 mg/kg, The group serving as the control got oral distilled water (2 mL/rat), while the standard group consumed oral loperamide (5 mg/kg). All rats were given castor oil (2 mL/rat) orally, which resulted in diarrhea 1 h after the standard and the extract was administered.

Each was housed separately in a cage topped with white paper to gather excrement. Hard and soft debris were counted over 6 h, and the onset of diarrhea was tracked (Mady et al. 2022). By applying subsequent formulas, the percentages of diarrhea and defecation inhibition were established (Megersa et al. 2023).

$$\begin{array}{l}{\% of inhibition of diarrhea }=\frac{\text{mean wet feces number of control group}-\text{ mean wet feces of treated group }}{mean \,wet\, feces \,number\, of\, control\, group}\times 100\\ {\% of inhibition of defecation }=\frac{\text{total feces number of control group}-\text{ total feces number of treated group }}{ total\, feces\, number \,of \,control\, group}\times 100\end{array}$$

#### Test of gastrointestinal motility in rats

After an 18-h fast, thirty rats were randomized into five groups (*n* = 6). Each rat was first given 1 mL of castor oil to induce diarrhea. An hour later, the three test groups were given 250, 500, and 1000 mg/kg of AME of *S. Japonica* fruit, the standard group was given oral loperamide (5 mg/kg), and the control group was given oral distilled water (2 mL/rat).

All rats were given charcoal meal (1 mL/rat) orally 1 h later. This meal was made up of 10% charcoal suspended in 5% gum acacia. The rats were sacrificed an hour after the charcoal meal was provided.

We measured the distance the charcoal traveled and calculated the intestinal transit percentage by dividing this distance by the total length of the small intestine (pylorus to cecum), as previously described (Kola-Mustapha et al. 2019). The subsequent calculation of intestinal transit and percentage of inhibition is detailed below:

$$\begin{array}{l}\text{Intestinal Transit }(\mathrm{IT}) =\frac{Distance\, Travelled \,by \,Charcoal \,Meal}{Total\,intestinal \,length}\times 100\\ {\% of Inhibition }=\frac{IT\,of \,the\, control\, group -IT\, of \,the \,treated\,group}{IT\, of \,the\, control\, group}\times 100\end{array}$$

#### Castor oil induces enterpooling in rats

Rats that had fasted overnight were placed into five groups (*n* = 6), just as in the two prior tests. Each rat was orally administered 2 ml of castor oil before being treated with the standard and tested extracts for 1 h. After two hours, the rats were euthanized, and their small intestines were removed after being tied with strings at both ends. The intestinal contents were extracted, and their volumes were determined using a graduated cylinder. The percentage inhibition by mean volume of intestinal content (MVIC) was calculated through the following formula (Megersa et al. 2023):

$$\text{Percentage inhibition of MVIC }=\frac{MVICC-MVICT}{MVICC}\times 100$$

where MVICT and MVICC, respectively, represent the mean intestinal content volumes of the test and control groups.

#### A measure of the antidiarrheal index (ADI)

The following formula was used to calculate the in vivo antidiarrheal index (ADI) based on data from each of the three tests (Andargie et al. 2022).

$$\mathrm{ADI}=\sqrt[3]{Dfreq\times Gmeq\times P \,freq}$$

$$\text{D freq}=\frac{mean\, diarrheal\, onset \,of\, treated \,group-mean\, diarrheal \,onset\, of \,control \,group}{mean \,diarrheal \,onset \,of \,control\, group}$$

D freq is the castor oil diarrheal trial-derived delay in the onset of defecation.

G meq is the reduction in gut meal travel found in the charcoal meal investigation.

P freq is the purging frequency determined by the decrease in wet stool from the castor oil-triggered diarrheal trial.

#### Investigation of statistics

The mean ± standard error of the mean (SEM) was used to convey findings of the in vivo study. One-way Analysis of Variance (ANOVA) and the Tukey–Kramer multiple-comparison test were used to assess statistical significance within groups. All analyses were performed using GraphPad Prism v8 (GraphPad Software, United States), and the level of significance was set at *p* < 0.05.

## Results

### Total phenolic and flavonoid content of the extract

The 80% methanolic extract of *S. japonica* fruits contained substantial levels of phenolic and flavonoid compounds.

#### Phenolic contents of the extract

The calibration curve for gallic acid demonstrated linearity between 12.5–200 µg/mL (R^2^ = 0.999), corresponding to **46.25 ± 2.86 mg GAE/g**.

#### Flavonoid contents of the extract

Similarly, the quercetin calibration curve was linear from 12.5–100 µg/mL (R2 = 0.999), yielding 22.18 ± 1.73 mg QE/g.

### Identification of isolated compounds

**Fig. 2:**
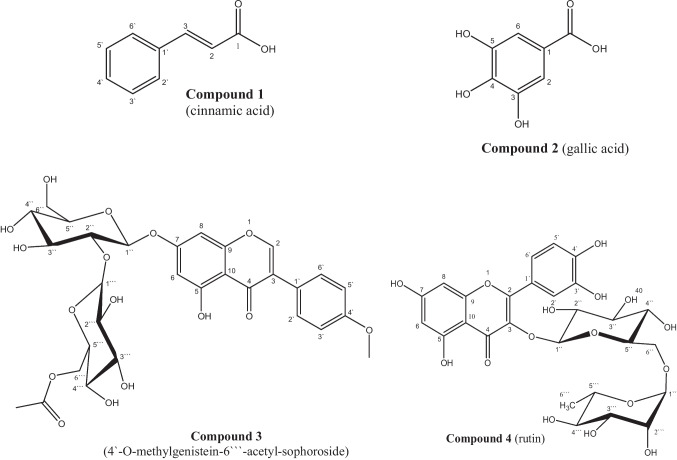

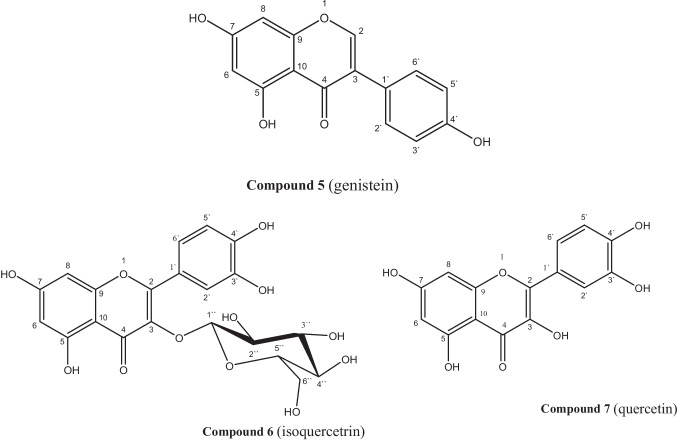
Structure of isolated compounds from *S. japonica* fruit AME

Chromatographic fractionation of the AME yielded seven polyphenolic compounds. Based on spectral data (^1^H -NMR, ^13^C -NMR) and CoPC with authentic standards, the isolated compounds were identified as **compound 1** (cinnamic acid), **compound 2** (gallic acid), **compound 4** (rutin), **compound 5** (genistein), **compound 6** (isoquercitrin), **compound 7** (quercetin), and **compound 3** a new compound identified as (4′-*O*-methylgenistein-6′′′-acetyl-sophoroside). (Fig. 2). Structural identification was supported by previous reports (Agrawal et al. 1989; Tomczyk et al. 2002; Abdelhady et al. 2015; Moharram et al. 2018; He et al. 2018; Mahmoud et al. 2001; Allam et al. 2012).

#### Spectra and NMR data of compounds 1,2, (4–7) are available as a supplementary file

**Identification of Compound 3** (new compound):

The UV–visible spectrum in MeOH (Figure S1) revealed two characteristic absorption maxima: a broad band at 260 nm (band II) and a low-intensity band at 330 nm (band I). This data coincides with a typical isoflavone structure (Mabry et al. 1970).

^**1**^**H NMR** (400 MHz, **DMSO-*d***_***6***_) (Figure S3), δ ppm 8.31 (1H, s, H-2), 6.5 (1H, brs, H-6), 6.8 (1H, brs, H-8), 7.5 (1H, d, *J* = 8.48 Hz, H-2՛/6՛), 7.2 (1H, d, *J* = 8.50 Hz, H-3^՛/^5՛), 5.1 (1H, d,* J* = 7.2 Hz H1^՛՛^), 4.8 (1H, d, *J* = 7.1 Hz H-1^՛՛՛^), 3.8 (3H, s, OCH_3_ at position 4^՛^), 3.2–4.6 (m, remaining of the two sugar protons). 2 (3H, s, CH_3_ of acetyl at position-6^՛՛՛^).

^**13**^**C NMR** (100 MHz, **DMSO-*d***_***6***_) (Figure S4), δ ppm 181.45 (C-4), 170 (carbonyl of acetyl group), 164.58 (C-7), 160.14 (C-5), 159.08 (C-4՛), 157.06 (C9), 154.11 (C-2), 122.50 (C-3), 122.39 (C-1՛), 129.72 (C-2՛/6՛), 114.62 (C-3՛/5՛), 104.11 (C-10), 103.79 (C-1՛՛՛), 103.61 (C-1՛՛), 100.05 (C-6), 94.91 (C-8), 81.48 (C-2՛՛), 74.9 (C-5՛՛), 74 (C-3՛՛՛), 73.5 (C-5՛՛՛), 73.3 (C-3՛՛), 73.2 (C-2՛՛՛), 70.7 (C-4՛՛), 70.08 (C-4՛՛՛), 65.56 (C-6՛՛՛), 61.56 (C-6՛՛), 56.33 (C-OCH_3_), 20.24 (C-acetyl group). Its -ve ESI–MS showed m/z at 649.057{M-H}^−^ (Figure S2).

The ^**1**^**H NMR** spectrum of compound 3 recorded in DMSO-d₆ (400 MHz, Figure S3) displayed signals characteristic of an isoflavone diglycoside structure. The singlet observed at δ 8.31 ppm (1H, s) was assigned to H-2 of the isoflavone nucleus, a diagnostic feature of genistein-type aglycones (He et al. 2018). The A₂X₂ spin coupling system appearing as two ortho-coupled doublets at δ 7.5 ppm (1H, d, *J* = 8.48 Hz, H-2′/6′) and δ 7.2 ppm (1H, d, *J* = 8.50 Hz, H-3′/5′) indicated a para-substituted B-ring, consistent with a 4′-substituted genistein moiety.

The two broad singlet signals at δ 6.5 ppm (1H, br s, H-6) and δ 6.8 ppm (1H, br s, H-8) correspond to the meta-coupled protons of ring A. Their downfield chemical shifts relative to free genistein are indicative of glycosylation at the C-7 hydroxyl group, supporting a 7-*O*-substituted genistein structure.

Two anomeric proton signals were clearly observed at δ 5.1 ppm (1H, d, J = 7.2 Hz, H-1″) and δ 4.8 ppm (1H, d, *J* = 7.1 Hz, H-1‴), each exhibiting coupling constants characteristic of β-configured glucopyranosyl units. The remaining sugar protons resonated as a multiplet in the range δ 3.2–4.6 ppm, confirming the presence of a diglucosyl moiety.

Additionally, the singlet at δ 3.8 ppm (3H, s) was assigned to a methoxy group attached to C-4′ of the B-ring. In comparison, the singlet at δ 2.0 ppm (3H, s) corresponded to a methyl group of an acetyl substituent, indicating acetylation of the sugar moiety. These spectral features are in good agreement with previously reported data for acetylated methoxy-substituted diglycosides (Mahmoud et al. 2001; Allam et al. 2012).

Overall, the ^1^H NMR data support the assignment of compound 3 as a methoxylated, acetylated 7-*O*-diglucosyl genistein derivative, consistent with literature reports and corroborated by complementary spectroscopic analyses.

The ^**13**^**C NMR** spectrum of compound 3 recorded in DMSO-d₆ (100 MHz, Figure S4) exhibited carbon resonances characteristic of an isoflavone diglycoside bearing methoxy and acetyl substituents. The downfield signal at δ 181.45 ppm was assigned to the conjugated carbonyl carbon (C-4) of the isoflavone nucleus, confirming the flavone-type skeleton. The presence of an additional carbonyl resonance at δ 170 ppm corresponded to the ester carbonyl of an acetyl group, indicating acetylation within the molecule. (Mahmoud et al. 2001; Allam et al. 2012).

The aromatic methine carbons of ring B appeared at δ 129.72 ppm (C-2′/6′) and δ 114.62 ppm (C-3′/5′), consistent with a para-substituted B-ring, while C-1′ resonated at δ 122.39 ppm. The remaining aromatic carbons of the isoflavone core were observed at δ 122.50 (C-3), 104.11 (C-10), 100.05 (C-6), and 94.91 ppm (C-8), further confirming the genistein-type aglycone.

The anomeric carbons of the two glucopyranosyl units were clearly observed at δ 103.61 ppm (C-1″) and δ 103.79 ppm (C-1‴), supporting the presence of a diglucosyl moiety. The remaining sugar carbons resonated in the δ 61.56–81.48 ppm region. Notably, the downfield shift of C-2″ at δ 81.48 ppm is diagnostic for an interglycosidic linkage and confirms a (1‴ → 2″) sophoroside-type connection between the two glucose units. Signals at δ 61.56 and 65.56 ppm were assigned to the primary alcohol carbons (C-6″ and C-6‴), while the remaining oxygenated methine carbons appeared between δ 70.08 and 74.90 ppm.

Additionally, the methoxy carbon was observed at δ 56.33 ppm, consistent with a 4′-O-methoxy substituent, while the methyl carbon of the acetyl group was observed at δ 20.24 ppm, further confirming sugar acetylation.

The negative-mode ESI–MS spectrum (Figure S2) showed a molecular ion peak at m/z 649.057 [M–H] ^⁻^, which is in agreement with the proposed molecular formula of an acetylated, methoxylated 7*-O-*diglucosyl genistein derivative. Taken together, the ^13^C NMR and mass spectrometric data strongly support the assigned structure of compound 3.

The **HSQC** spectrum (Figure S6) enabled unambiguous assignment of all protonated carbons through direct one-bond (^1^*J*_CH) correlations. A distinct cross-peak was observed between the singlet proton at δ H 8.31 (1H, s, H-2) and the corresponding carbon at δ C 154.11 (C-2), confirming the assignment of H-2 on the isoflavone nucleus.

Correlation between the methyl protons of the acetyl group (δ 2.0 ppm, 3H) at position 6^’’’^ and its corresponding carbon (δ 20.24 ppm) in the HSQC spectrum, supporting the presence of an acetyl substituent on the sugar moiety. In addition, the methoxy group showed a characteristic cross-peak between δ H 3.80 (3H, s, OCH₃) and δ C 56.33, confirming the presence of a methoxy substituent.

All anomeric protons of the two sugar units showed well-resolved HSQC correlations with their respective anomeric carbons, allowing confident identification of the glycosidic units and their protonated carbons. Collectively, the HSQC data provided definitive assignments for all proton-bearing carbons and were fully consistent with the proposed structure.

Long-range ^*2*^*J*_CH and ^*3*^*J*_CH correlations observed in the **HMBC** spectrum (Figure S5) provided key evidence for the substitution pattern and interglycosidic linkages. A diagnostic HMBC correlation was observed between the methoxy protons (δ H 3.80) and the aromatic carbon at δ C 159.08 (C-4′), confirming methoxylation at position C-4′ of ring B.

The glycosylation site was established by the presence of HMBC cross-peaks between the anomeric protons at δ H 5.10 (1H, d, J = 7.2 Hz, H-1′′) and δ H 4.80 (1H, d, J = 7.1 Hz, H-1′′′) with the aglycone carbon at δ C 164.58 (C-7), confirming *O*-glycosylation at C-7 of the isoflavone structure.

The interglycosidic linkage was confirmed by a clear HMBC correlation between the anomeric proton of the terminal sugar (δ H 4.80, H-1′′′) and the inner sugar carbon at δ C 81.48 (C-2′′), establishing a β-D-glucopyranosyl-(1′′′ → 2′′)-β-D-glucopyranoside linkage.

Furthermore, the position of the acetyl group was supported by an HMBC correlation between the acetyl carbonyl-bearing sugar carbon at δ C 65.56 (C-6′′′) and the acetyl methyl protons (δ H 2.00), confirming acetylation at C-6′′′ of the terminal glucose unit.

Although NOESY correlations involving the acetyl methyl group were not observed, the assignment of the acetyl substituent is unambiguously supported by characteristic ^1^H and ^13^C chemical shifts, key HMBC correlations, and mass spectrometric data.

The HMBC spectrum displays multiple or overlapping cross-peaks, which can be attributed to conformational flexibility and/or minor tautomeric forms in solution. Such behavior is commonly observed in polyfunctionalized flavonoid glycosides bearing multiple hydroxyl, methoxy, and acetyl substituents. Despite this spectral complexity, all diagnostic long-range correlations required to establish the substitution pattern and connectivity are clearly observed and are fully consistent with the proposed structure.

Based on comprehensive spectroscopic analysis, including HSQC, HMBC, ^1^H NMR, ^13^C NMR, and MS data, compound 3 was unambiguously identified as **4′-*O*-methylgenistein-7-*O*−6′′′-*O*-acetyl-*β*-D-glucopyranosyl-(1′′′ → 2′′)-*β*-D-glucopyranoside** (4′-*O*-methylgenistein-6′′′-acetyl-sophoroside). To the best of our knowledge, this compound is reported here for the first time.

### Antioxidant activity

**Fig. 3:**
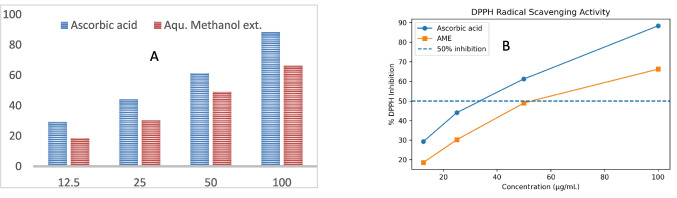
Antioxidant activity of *S. japonica* fruit AME (**A**) and DPPH radical scavenging activity of ascorbic acid and AME of *S. japonica* at different concentrations. Values represent mean % inhibition. The dashed line indicates 50% inhibition used for IC₅₀ determination (**B**)

The AME demonstrated dose-dependent capacity to scavenge free radicals in the DPPH test, at 100 µg/mL. The DPPH assay is a widely used method for assessing the free radical scavenging ability of natural and synthetic antioxidants. The observed concentration-dependent increase in DPPH inhibition for both ascorbic acid and AME confirms their ability to donate hydrogen atoms or electrons to neutralize DPPH radicals. Ascorbic acid showed superior antioxidant activity, which is consistent with its well-established role as a potent free radical scavenger. Its low IC₅₀ value (33.6 µg/mL) reflects its high efficiency in reducing DPPH radicals even at low concentrations. AME exhibited appreciable antioxidant activity, although its IC₅₀ value (53.0 µg/mL) was higher than that of ascorbic acid, indicating comparatively lower radical scavenging strength. This activity may be attributed to the presence of bioactive phytochemicals such as phenolic compounds and flavonoids, which are known to contribute to antioxidant properties through redox mechanisms. The extract produced 66.17% inhibition, compared with 88.37% for ascorbic acid at the same concentration, indicating preliminary antioxidant potential (Table 1 and Fig. 3).

**Table 1:** DPPH radical scavenging activity of ascorbic acid and AME **of *S. japonica*** at different concentrations

Conc. Ascorbic acid ug/ml	Conc. AME ug/ml	% of inhibition Ascorbic acid	% of inhibition AME	SD (Ascorbic acid)	SD (AME)
12.5	12.5	29.282	18.54	0.27	1.7
25	25	44.10408102	30.24	0.48	2.37
50	50	61.1660079	48.97895256	0.31	1.69
100	100	88.367	66.17259881	0.73	2.5778

### In vivo study

#### Analysis of acute toxicity

At the highest dose of extract tested in the acute study (5 g/kg), there was no indication of animal death or behavioral alterations. Therefore, the AME at doses of 250, 500, and 1,000 mg/kg seemed safe.

#### Impact of *S. japonica* fruit aqueous methanol extract (AME) on rats’ diarrhea triggered by caster oil

Treatment with standard and the AME at 250, 500, and 1000 mg/kg revealed a significant delay in the onset of diarrhea by onefold, 11%, 51.6%, and onefold, respectively, compared with the control group. The substantial decline in both the total number of feces and their wetness at the three doses confirmed the extract’s antidiarrheal capabilities. They mitigate diarrhea by 36.4%, 61.4%, and 86.4%, in that order. The optimal effect was observed at 1000 mg/kg, which is equivalent to the standard inhibitory effect. Furthermore, the extract inhibited defecation by 32, 53, and 83.4% at the three doses. Additionally, 1000 mg/kg produced the most significant effect, which is not statistically distinguishable from the standard inhibitory influence (Table 2).

**Table 2:** Impact of *S. japonica* fruit AME on diarrhea caused by castor oil

Group	Dose	Onset of diarrhea (min)	Total number of wet feces (TNWF)	Total number of feces	%of diarrhea inhibition	%of defecation inhibition
Control		91.67 ± 1.93	7.34 ± 0.34	11 ± 0.37	-	-
Loperamide	5 mg	189.7 ± 2.95^a^	0.834 ± 0.31^a^	2 ± 0.26^a^	88.7%	81.8%
*S. japonica* fruit AME	250	101.8 ± 2.12^b^	4.67 ± 0.34^a,b^	7.5 ± 0.43^a,b^	36.4%	32%
	500	139 ± 3.56^a,b,c^	2.83 ± 0.3^a,b,c^	5.2 ± 0.31^a,b,c^	61.4%	53%
	1000	185 ± 2.35^a,c,d^	1 ± 0.26^a,c,d^	1.83 ± 0.31^a,c,d^	86.4%	83.4%

Values are stated in the form of mean ± SE (*n* = 6). Significant findings were identified relative to the control group (^a^), the standard group (^b^), the aqueous methanol extract (AME) of *S.japonica* (250 mg/kg) group (^c^), and the AME of *S.japonica* (500 mg/kg) group (^d^)

#### Impact of *S. japonica* fruit aqueous methanol extract (AME) on a rat’s gastrointestinal motility test

One measure of gastrointestinal motility is intestinal transit (IT) after a charcoal meal. Our findings showed that the three AME dosages tested significantly reduced the distance of the charcoal meal. Intestinal transit was significantly reduced by 43.5%, 22.6%, 30.12%, and 42%, respectively, following treatment with the standard and the AME; the most significant inhibition was observed at 1000 mg/kg (Table 3).

**Table 3:** Impact of *S. japonica* fruit AME on gastrointestinal motility caused by castor oil

Group	Dose	Total Intestinal Length (TIL) cm	Distance Travelled by Charcoal Meal (DTCM) cm	Intestinal Transit (IT) cm	%of inhibition
Control		90.29 ± 0.72	78.67 ± 1.49	87.15 ± 1.66	-
Loperamide	5 mg	90.43 ± 0.99	44.5 ± 1.48^a^	49.23 ± 1.63^a^	43.5%
*S. japonica* fruit AME	250	90 ± 1	60.67 ± 0.88^a,b^	67.42 ± 0.98^a,b^	22.6%
	500	89.86 ± 0.89	54.83 ± 1.078^a,b,c^	60.9 ± 1.2^a,b,c^	30.12%
	1000	89.29 ± 0.97	45.17 ± 1.2^a,c,d^	50.6 ± 1.4^a,c,d^	42%

Values are stated in the form of mean ± SE (*n* = 6). Significant findings were identified relative to the control group (^a^), the standard group (^b^), the aqueous methanol extract (AME) of *S.japonica* (250 mg/kg) group (^c^), and the AME of *S.japonica* (500 mg/kg) group (^d^)

#### Impact of *S. japonica* fruit aqueous methanol extract (AME) on enteropooling caused by castor oil

To quantify the AME’s effect on castor oil-induced enteropooling, we measured the mean volume of intestinal content (MVIC) and the percentage of inhibition. The MVIC was significantly reduced across all active groups, yielding 73.2% inhibition (standard) and 29.2%, 46%, and 70% inhibition for the three extract doses, respectively. Maximum inhibition was achieved at 1000 mg/kg (Table 4).

**Table 4:** Impact of *S. japonica* fruit AME on enteropooling caused by castor oil

Group	Dose	Mean volume of intestinal content (MVIC) ml	% of inhibition
Control		4.16 ± 0.16	-
Loperamide	5 mg	1.12 ± 0.09^a^	73.2%
*S. japonica* fruit AME	250	2.95 ± 0.07^a,b^	29.2%
	500	2.25 ± 0.08^a,b,c^	46%
	1000	1.25 ± 0.07^a,c,d^	70%

Values are stated in the form of mean ± SE (*n* = 6). Significant findings were identified relative to the control group (^a^), the standard group (^b^), the aqueous methanol extract (AME) of *S. japonica* (250 mg/kg) group (^c^), and the AME of *S. japonica* (500 mg/kg) group (^d^)

#### Impact of *S. japonica* fruit aqueous methanol extract (AME) on the in vivo antidiarrheal index (ADI)

At 250, 500, and 1000 mg/kg of the extract, the ADI values were 20.9, 45.7, and 71.6, respectively; in contrast, the standard showed an ADI of 74.4. With 1000 mg/kg demonstrating the most potent antidiarrheal activity, these findings demonstrated the AME’s considerable antidiarrheal potential (Table 5).

**Table 5:** The impact of *S. japonica* fruit AME on the in vivo antidiarrheal index (ADI)

Group	Dose	D freq	Gmeg	Pfreq	ADI
Control		-	-	-	-
Loperamide	5 mg	106.2	43.5	88.7	74.4
***S. japonica*** fruit AME	250	11.1	22.6	36.4	20.9
	500	51.6	30.12	61.4	45.7
	1000	101.1	42	86.4	71.6

## Discussion

Castor oil was used as a stimulant laxative in three diarrhea models to evaluate the antidiarrheal activity in vivo*.* The pharmacologically active ingredient of castor oil is ricinoleic acid, which is generated by the lipase enzyme and found in the upper portion of the small intestine. Therefore, inhibition of this process implies that the extract may suppress prostaglandin-mediated hypersecretion or reduce intestinal motility (Kulkarni and Pandit 2005). The prostanoid EP3 receptor is bound and activated by ricinoleic acid, which also promotes the synthesis of endogenous prostaglandins from intestinal arachidonic acid. The prostaglandins can advance, change the flow of water and salts into and out of the intestinal lumen, alter gastrointestinal motility, and have a laxative effect (Mady et al. 2023). Castor oil increases prostaglandin production, which stimulates fluid output (Pierce et al*.* 1971). As a result, the use of castor oil to induce diarrhea is justified in this study, as it mimics the pathophysiology of diarrhea.

According to other studies, the secondary metabolites of traditional medicinal herbs, such as tannins and flavonoids, are usually associated with their ability to prevent diarrhea (Mady et al. 2023). The B-ring catechol scaffold and C-4 carbonyl group, as well as the quantity and location of hydroxyl groups and the kind of substitution, are structural characteristics of flavonoids that determine their antioxidant properties. By scavenging free radicals and/or chelating metal ions, flavonoids collectively mediate their antioxidant actions. Considering the previously provided information, we assessed the antioxidant potential of AME. Our data revealed that AME exerted significant antioxidant activity. Accordingly, the antioxidant properties of AME correlated with its high content of phenolic compounds, including flavonoids and tannins (Mady et al. 2022). Castor oil causes oxidative damage by producing ROS and stimulating nitric oxide release, which causes inflammation, all this can potentiate diarrhea by inducing damage to the gastrointestinal mucosa. It also causes Ca2 + to leak into the cytoplasm by blocking the Ca2 +/ATPase pump, which destabilizes the endoplasmic reticulum, leading to increased secretion and intestinal motility (Brinsi et al. 2024). Polyphenol compounds play a major role in absorbing and neutralizing ROS free radicals and decomposing peroxides, which causes a decrease in diarrhea triggered by castor oil (Ladjimi et al. 2023). Therefore, AME has antidiarrheal activity owing to its antioxidant capacity against castor oil. Our results are in accordance with Lad Jimii et al. (2023) and Brinsi et al. (2024). Moreover, it was found that plants used to treat diarrhea have been found to act through various mechanisms, including changes in peristaltic index, reductions in stomach motility, and antiparasitic effects (Özbilgin et al. 2013). According to our findings, AME significantly improved all parameters assessed, including the amount of wet, watery, and total excrement, as well as the onset of diarrhea. This suggests that AME works against diarrhea by inhibiting the secretion of certain chemicals.

To further support the extract’s antidiarrheal action, motility and pooling tests were evaluated. Our findings, based on the experimental data (observed pharmacological effects; the castor oil-triggered enterpooling test), showed that *S. japonica* AME significantly reduced intestinal content volume to nearly the same level as the standard at the maximum dosage, by preventing the reabsorption of water and electrolytes. The AME increased the reabsorption of water and electrolytes; it stopped enterpooling and gastrointestinal hypersecretion, alternatively, by blocking the buildup of intestinal fluid, which may once more be linked to their polyphenolic composition. Furthermore, due to decreased gastrointestinal motility, the charcoal meal strategy was evaluated to track the transit of gastrointestinal material and to elucidate additional antidiarrheal mechanisms (Qnais et al*.* 2005; Ezekwesili et al. 2010). The recent findings indicated that decreasing gastrointestinal motility with charcoal meals resulted in AME significantly hindering intestinal transit, increasing intestinal content and passage time. Therefore, it was suggested that all intestinal sections are affected by the antimotility characteristics of AME (Islam et al. 2013). The extract’s relative effectiveness in treating diarrhea was indicated by the ADI value (Zayede et al*.* 2020). Treating diarrhea became more effective as the ADI values increased. According to the current study, 1000 mg/kg of the AME resulted in an ultimate ADI of 71.6, demonstrating that this dose is highly effective in treating diarrhea.

Additionally, as previously reported in the literature (hypothesized mechanisms), plants containing quercetin (**Compound 7**) can exhibit antidiarrheal effects primarily due to their anti-inflammatory and antihistamine properties (Mady et al. 2022). By preventing the gastrointestinal system from releasing acetylcholine, quercetin stops diarrhea. (Lutterodt 1989; Perrucci et al. 2006). Flos *S. Japonica* contains triterpenoid saponins, betulin, sophoradiol, glucose, glucuronic acid, as well as flavonoids and tannins. Additionally, the presence of tannins is known to denature intestinal mucosal proteins. They decrease secretion because they are more resistant to chemical alterations. Furthermore, the anti-spasmodic properties of tannins, which reduce Ca^2+^ influx or increase Ca^2+^ outflow to restrict secretion and intestinal motility, confer antimotility activity. Earlier studies showed that tannins can bind to proteins, forming complexes that precipitate intestinal mucosal proteins, thereby diminishing intestinal secretion and peristaltic movements due to their resistance to chemical change (Kumar and Upadhyaya 2012). Additionally, the presence of gallic acid (**Compound 2**) may increase anti-diarchal action because it inhibits H^+^K^+^-ATPase activity, which has anti-secretory activity (Chen et al. 2006). *S. japonica* was abundant in flavonoids, which emphasizes preliminary pharmacological activity to impede intestinal motility and the synthesis of water and electrolytes by interfering with prostaglandins’ auto-acid and activity (Tiwari et al. 2011). Thus, the interaction among the extract’s various ingredients is thought to be responsible for the crude extract’s effectiveness, leading to good activity. As an alternative, the extract’s effectiveness may be influenced by the combined effects of the various ingredients. This result supports the use of the extract in ethnomedicine for the treatment of diarrhea.

## Limitations

This study has several limitations that should be considered. First, the proposed mechanistic explanations, including inhibition of prostaglandin-mediated secretion, reduced intestinal motility, and protein precipitation by tannins, were not directly experimentally validated. Second, the study relied on crude extract testing without quantifying individual bioactive constituents, limiting precise mechanistic interpretation. Third, advanced analytical techniques, such as prostaglandin quantification, smooth muscle contractility assays, or molecular pathway analyses, were not employed, which could have provided deeper mechanistic insight. In the present study, DPPH was employed as a preliminary screening tool due to its simplicity and widespread use. To avoid overinterpretation, we have revised the manuscript to moderate the antioxidant claims and explicitly acknowledge the limitations of using a single assay. We have also indicated that additional antioxidant assays will be included in future studies. We explicitly acknowledged the absence of bioactivity-guided fractionation as a limitation. Finally, the sample size (*n* = 6 per group) is relatively small, which may modestly limit statistical power and generalizability. Future studies incorporating mechanistic assays, purified compounds, and larger cohorts are warranted to confirm and extend these findings.

## Conclusion

**Fig. 4:**
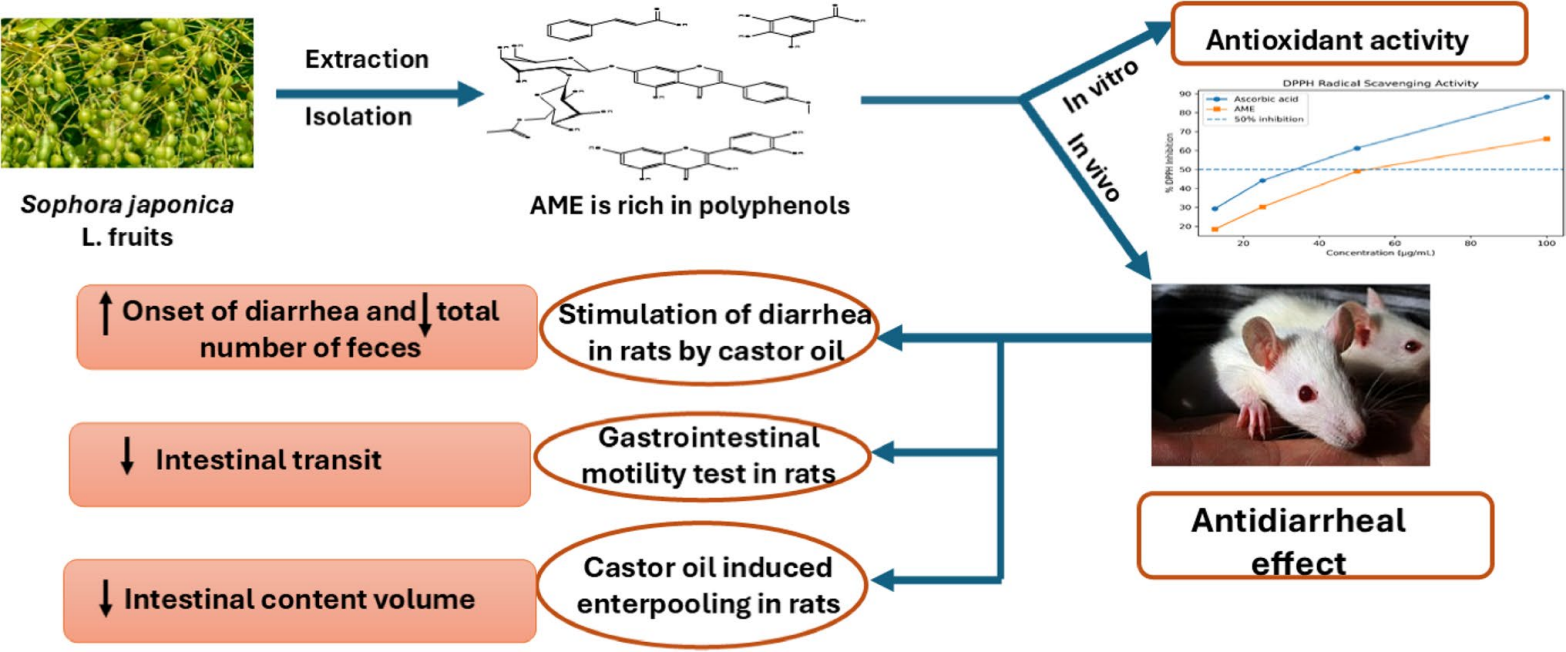
Effect of aqueous methanol extract of *S. Japonica* fruit on antioxidant activity and antidiarrheal effect

The study’s findings demonstrated the separation and identification of seven phenolic components of *S. Japonica* fruits AME. The AME demonstrated both antidiarrheal and antioxidant properties. This result supports the use of the extract in ethnomedicine for the treatment of diarrhea. Our work suggests additional clinical research on *S. Japonica* as a palliative natural remedy for diarrhea, given the pressing need to discover new adjuvant and supplementary herbal drugs for numerous illnesses (Fig. 4).

## Supplementary Information

Below is the link to the electronic supplementary material.

Supplementary file1 (DOCX 1430 KB)

## Data Availability

The data supporting the findings of this study are included within the article and its supplementary materials.
