# Supplementary material for: Phytochemical profiling and antidiarrheal activity of Sophora japonica L. fruit extract
Source: Saudi Pharm J. 2026 Apr 16;34(2):21. doi: 10.1007/s44446-026-00081-3 (PMC13086991; doi:10.1007/s44446-026-00081-3)
Supplement: Supplementary file 1 — Supplementary file1 (DOCX 1430 KB) [file 44446_2026_81_MOESM1_ESM.docx]

**Compounds 1 and 2**: They are **tentatively identified** according to their chromatographic characters and compared with reference samples.

**Compound 3**:

The UV-visible spectrum in MeOH (Figure S1), revealed two characteristic absorption maxima at 260 nm band II and 330 nm with low intensity band I. This data is coinciding with a typical isoflavone structure (Mabry *et al.,* 1970).

**^1^H NMR** (400 MHz, **DMSO-*d_6_***) (Figure S3), δ ppm 8.31 (1H, s, H-2), 6.5 (1H, brs, H-6), 6.8 (1H, brs, H-8), 7.5 (1H, d, *J* = 8.48 Hz, H-2՛/6՛), 7.2 (1H, d, *J* = 8.50 Hz, H-3^՛/^5՛), 5.1 (1H, d, *J* = 7.2 Hz H1^՛՛^ ), 4.8 (1H, d, *J* = 7.1 Hz H-1^՛՛՛^ ), 3.8 (3H, s, OCH_3_ at position 4^՛^ ), 3.2-4.6 (m, remaining of the two sugar protons). 2 (3H, s, CH_3_ of acetyl at position-6^՛՛՛^).

**^13^C NMR** (100 MHz, **DMSO-*d_6_***) (Figure S4), δ ppm 181.45 (C-4), 170 (carbonyl of acetyl group), 164.58 (C-7), 160.14 (C-5), 159.08 (C-4՛), 157.06 (C9), 154.11 (C-2), 122.50 (C-3), 122.39 (C-1՛), 129.72 (C-2՛/6՛), 114.62 (C-3՛/5՛), 104.11 (C-10), 103.79 (C-1՛՛՛), 103.61 (C-1՛՛), 100.05 (C-6), 94.91 (C-8), 81.48 (C-2՛՛), 74.9 (C-5՛՛), 74 (C-3՛՛՛), 73.5 (C-5՛՛՛), 73.3 (C-3՛՛), 73.2 (C-2՛՛՛), 70.7 (C-4՛՛), 70.08 (C-4՛՛՛), 65.56 (C-6՛՛՛), 61.56 (C-6՛՛), 56.33 (C-OCH_3_), 20.24 (C-acetyl group). Its -ve ESI-MS showed m/z at 649.057{M-H}^-^ (Figure S2).

The **^1^H NMR** spectrum of compound 3 recorded in DMSO-d₆ (400 MHz, Figure S3) displayed signals characteristic of an isoflavone diglycoside structure. The singlet observed at δ 8.31 ppm (1H, s) was assigned to H-2 of the isoflavone nucleus, which is diagnostic for genistein-type aglycones (He *et al.,* 2018). The A₂X₂ spin coupling system appearing as two ortho-coupled doublets at δ 7.5 ppm (1H, d, *J =* 8.48 Hz, H-2′/6′) and δ 7.2 ppm (1H, d, *J* = 8.50 Hz, H-3′/5′) indicated a para-substituted B-ring, consistent with a 4′-substituted genistein moiety.

The two broad singlet signals at δ 6.5 ppm (1H, br s, H-6) and δ 6.8 ppm (1H, br s, H-8) correspond to the meta-coupled protons of ring A. Their downfield chemical shifts relative to free genistein are indicative of glycosylation at the C-7 hydroxyl group, supporting a 7-*O*-substituted genistein structure.

Two anomeric proton signals were clearly observed at δ 5.1 ppm (1H, d, J = 7.2 Hz, H-1″) and δ 4.8 ppm (1H, d, *J* = 7.1 Hz, H-1‴), each exhibiting coupling constants characteristic of β-configured glucopyranosyl units. The remaining sugar protons resonated as a multiplet in the range δ 3.2–4.6 ppm, confirming the presence of a diglucosyl moiety.

Additionally, the singlet at δ 3.8 ppm (3H, s) was assigned to a methoxy group attached at C-4′ of the B-ring, while the singlet at δ 2.0 ppm (3H, s) corresponded to a methyl group of an acetyl substituent, indicating acetylation of the sugar moiety. These spectral features are in good agreement with previously reported data for acetylated methoxy-substituted diglycosides (Mahmoud *et al.,* 2001; Allam *et al.,* 2012).

Overall, the ^1^H NMR data support the assignment of compound 3 as a methoxylated, acetylated 7-*O*-diglucosyl genistein derivative, consistent with literature reports and corroborated by complementary spectroscopic analyses.

The **¹³C NMR** spectrum of compound 3 recorded in DMSO-d₆ (100 MHz, Figure S4) exhibited carbon resonances characteristic of an isoflavone diglycoside bearing methoxy and acetyl substituents. The downfield signal at δ 181.45 ppm was assigned to the conjugated carbonyl carbon (C-4) of the isoflavone nucleus, confirming the flavone-type skeleton. The presence of an additional carbonyl resonance at δ 170 ppm corresponded to the ester carbonyl of an acetyl group, indicating acetylation within the molecule. (Mahmoud *et al.,* 2001; Allam *et al.,* 2012).

The aromatic methine carbons of ring B appeared at δ 129.72 ppm (C-2′/6′) and δ 114.62 ppm (C-3′/5′), consistent with a para-substituted B-ring, while C-1′ resonated at δ 122.39 ppm. The remaining aromatic carbons of the isoflavone core were observed at δ 122.50 (C-3), 104.11 (C-10), 100.05 (C-6), and 94.91 ppm (C-8), further confirming the genistein-type aglycone.

The anomeric carbons of the two glucopyranosyl units were clearly observed at δ 103.61 ppm (C-1″) and δ 103.79 ppm (C-1‴), supporting the presence of a diglucosyl moiety. The remaining sugar carbons resonated in the region δ 61.56–81.48 ppm. Notably, the downfield shift of C-2″ at δ 81.48 ppm is diagnostic for an interglycosidic linkage and confirms a (1‴→2″) sophoroside-type connection between the two glucose units. Signals at δ 61.56 and 65.56 ppm were assigned to the primary alcohol carbons (C-6″ and C-6‴), while the remaining oxygenated methine carbons appeared between δ 70.08 and 74.90 ppm.

Additionally, the methoxy carbon was identified at δ 56.33 ppm, consistent with a 4′-*O*-methoxy substituent, while the methyl carbon of the acetyl group appeared at δ 20.24 ppm, further confirming sugar acetylation.

The negative-mode ESI-MS spectrum (Figure S2) showed a molecular ion peak at m/z 649.057 [M–H] ^⁻^, which is in agreement with the proposed molecular formula of an acetylated, methoxylated 7*-O-*diglucosyl genistein derivative. Taken together, the ¹³C NMR and mass spectrometric data strongly support the assigned structure of compound 3.

The **HSQC** spectrum (Figure S6) enabled unambiguous assignment of all protonated carbons through direct one-bond (^1^*J*_CH) correlations. A distinct cross-peak was observed between the singlet proton at δ H 8.31 (1H, s, H-2) and the corresponding carbon at δ C 154.11 (C-2), confirming the assignment of H-2 on the isoflavone nucleus.

Correlation between the methyl protons of the acetyl group (δ 2.0 ppm, 3H) at position 6^׳׳׳^ and its corresponding carbon (δ 20.24 ppm) in the HSQC spectrum, supporting the presence of an acetyl substituent on sugar moiety. In addition, the methoxy group showed a characteristic cross-peak between δ H 3.80 (3H, s, OCH₃) and δ C 56.33, confirming the presence of a methoxy substituent.

All anomeric protons of the two sugar units showed well-resolved HSQC correlations with their respective anomeric carbons, allowing confident identification of the glycosidic units and their protonated carbons. Collectively, the HSQC data provided definitive assignments for all proton-bearing carbons and were fully consistent with the proposed structure.

Long-range *^2^J*_CH and *^3^J*_CH correlations observed in the **HMBC** spectrum (Figure S5) provided key evidence for the substitution pattern and interglycosidic linkages. A diagnostic HMBC correlation was observed between the methoxy protons (δ H 3.80) and the aromatic carbon at δ C 159.08 (C-4′), confirming methoxylation at position C-4′ of ring B.

The glycosylation site was established by the presence of HMBC cross-peaks between the anomeric protons at δ H 5.10 (1H, d, J = 7.2 Hz, H-1′′) and δ H 4.80 (1H, d, J = 7.1 Hz, H-1′′′) with the aglycone carbon at δ C 164.58 (C-7), confirming *O*-glycosylation at C-7 of the isoflavone structure.

The interglycosidic linkage was confirmed by a clear HMBC correlation between the anomeric proton of the terminal sugar (δ H 4.80, H-1′′′) and the inner sugar carbon at δ C 81.48 (C-2′′), establishing a β-D-glucopyranosyl-(1′′′→2′′)-β-D-glucopyranoside linkage.

Furthermore, the position of the acetyl group was supported by an HMBC correlation between the acetyl carbonyl-bearing sugar carbon at δ C 65.56 (C-6′′′) and the acetyl methyl protons (δ H 2.00), confirming acetylation at C-6′′′ of the terminal glucose unit.

Although NOESY correlations involving the acetyl methyl group were not observed, the assignment of the acetyl substituent is unambiguously supported by characteristic ^1^H and ^13^C chemical shifts, key HMBC correlations, and mass spectrometric data.

The HMBC spectrum displays multiple or overlapping cross-peaks, which can be attributed to conformational flexibility and/or minor tautomeric forms in solution. Such behavior is commonly observed in polyfunctionalized flavonoid glycosides bearing multiple hydroxyl, methoxy, and acetyl substituents. Despite this spectral complexity, all diagnostic long-range correlations required to establish the substitution pattern and connectivity are clearly observed and are fully consistent with the proposed structure.

On the basis of comprehensive spectroscopic analysis, including HSQC, HMBC, ^1^H NMR, ^13^C NMR, and MS data, compound 3 was unambiguously identified as **4′-*O*-methylgenistein-7-*O*-6′′′-*O*-acetyl-*β*-D-glucopyranosyl-(1′′′→2′′)-*β*-D-glucopyranoside** (4′-*O*-methylgenistein-6′′′-acetyl-sophoroside). To the best of our knowledge, this compound is reported here for the first time.

**Compound 4**:

**^1^H NMR** (400 MHz, **DMSO-*d_6_***) (**Figure S8):** δ ppm 7.55 (1H, dd, *J*= 8.38, 2.19 Hz, H-6`), 7.53 (1 H, d, *J*= 2.09 Hz, H-2`), 6.85 (1H, d, *J*= 8.35 Hz, H-5`), 6.39 (1H, d, *J*= 1.93 Hz, H-8), 6.18 (1H, d, *J*= 2.02 Hz, H- 6), 5.33 (1H, d, *J*= 7.26 Hz, H-1``), 4.40 (1H, brs, H-1```), 3.70 (1H, d, *J*= 10.61 Hz, H-6``), 3.43-3.06 (m, remaining sugar protons), 0.99 (3 H, d, *J*= 6.20 Hz, H-6```). **^13^C NMR** (100 MHz, **DMSO-*d_6_***) **(Figure S9):** δ ppm 177.72 (C-4), 166.39 (C-7), 161.68 (C-5), 157.30 (C-2), 157.14 (C-9), 149.54 (C-4`),145.64 (C-3`), 133.71 (C-3), 122.05 (C-6`), 121.40 (C-1`), 116.80 (C-2`), 115.96 (C-5`), 103.85 (C-10), 101.93 (C-1``), 101.35 (C-1```), 99.62 (C-6), 94.34 (C-8), 77.02 (C-3``), 76.37 (C-5``), 74.60 (C-2``), 72.43 (C-4```), 71.05 (C-3```), 70.87 (C-2```), 70.44 (C-4``), 68.81 (C-5```), 67.46 (C-6``), 18.32 (C-6```).

**Compound 5:** ^1^H NMR (400 MHz, DMSO-d6) (**Figure S11),** δ ppm 8.31 (1H, s, H-2), 6.21 (1H, brs, H-6), 6.38 (1H, brs, H-8), 7.46 (1H, d, J = 8.48 Hz, H-2՛/6՛), 6.95 (1H, d, J = 8.50 Hz, H-3՛/5՛).**^13^C NMR** (100 MHz, DMSO-d6) (Figure S12), δ ppm 181.36 (C-4), 163.69 (C-7), 160.65 (C-5), 159.79 (C4՛), 157.49 (C-9), 153.83 (C-2), 128.77 (C-2՛/6՛), 123.04 (C-3), 122.91 (C-1`), 115.14 (C-3՛/5՛), 104.85 (C-10), 100.05 (C-6), 94.47 (C-8). Its -ve ESI-MS showed m/z at 269.074{M-H}^-^ **(Figure S10).** Despite the complexity in the ^1^H NMR spectrum, the structural assignment is confirmed by MS and supported by **^13^C NMR** spectroscopic data.

**Compound 6**: **^1^H NMR** (400 MHz, DMSO-*d_6_*) **(Figure S13),** δ ppm 7.59 (1 H, dd, *J* = 8.38, 2.19 Hz, H-6`), 7.57 (1 H, d, *J* = 2.09 Hz, H-2`), 6.84 (1 H, d, J= 9.05 Hz, H-5`), 6.40 (1 H, d, *J* = 1.90 Hz, H-8), 6.20 (1 H, d, *J* = 1.86 Hz, H-6), 5.47 (1 H, d, *J*= 7.27 Hz, H-1``), 3.70 (2 H, d, *J* = 11.69 Hz, H-6``), 3.35- 3.08 (m, remaining of sugar protons). **^13^C NMR** (100 MHz, DMSO-d6) **(Figure S14),** δ ppm 178.00(C-4), 164.69 (C-7), 161.81 (C-5), 156.88 (C-2), 156.72 (C-9), 149.02 (C-4`),146.42 (C-3`), 133.87 (C-3), 122.16 (C-6`), 121.72 (C-1`), 116.75 (C-2`), 115.76 (C-5`), 104.53 (C-10), 101.39 (C-1``), 99.21 (C-6), 94.06 (C-8), 78.15 (C-5``), 77.06 (C-3``), 74.65 (C-2``), 70.49 (C-4``), 61.53 (C-6``).

**Compound 7**: **^1^H NMR** (400 MHz, DMSO-*d_6_*) **(Figure S15),** δ ppm 7.69 (1 H, d, *J*= 2.02 Hz, H-2`), 7.55 ( H, dd, *J*= 8.51, 2.05 Hz, H-6`), 6.89 (1 H, d, *J*= 8.52 Hz, H-5`), 6.41 (1 H, d, *J*= 1.95 Hz, H-8), 6.19 (1 H, d, *J*= 1.86 Hz, H-6). **^13^C APT** (100 MHz, DMSO-*d_6_*) **(Figure S16),** δ ppm 176.40 (C-4), 164.44 (C-7), 161.28 (C-5), 156.69 (C-9), 148.25 (C-4`), 147.35 (C-2), 145.61 (C-3`), 136.30 (C-3), 122.52 (C-1`), 120.54 (C-6`), 116.17 (C-2`), 115.63 (C-5`),103.58 (C-10), 98.74 (C-6), 93.91 (C-8).

**Fig. S1. UV-visible data of compound 3**

**Fig. S2. ESI-MS spectrum of compound 3**

**Fig. S3. ^1^H NMR spectrum of Compound 3**

**Fig. S4. ^13^C NMR spectrum of compound 3**

**Fig.S5. HMBC spectrum of compound 3**

**HSQC spectrum of compound 3**

**Fig. S6. HSQC spectrum of compound 3**

**NOESY spectrum of compound 3**

**Fig. S7. NOESY spectrum of compound 3**

**Fig. S8. ^1^H NMR spectrum of Compound 4 (400 MHz, DMSO-d6)**

**Fig. S9. ^13^C NMR spectrum of compound 4 (100MHz, DMSO-*d*_6_)**

ESI-MS of compound 5

**Fig. S10. ESI-MS spectrum of compound 5**

**Fig. S11. ^1^H NMR spectrum of Compound 5 (400 MHz, DMSO-*d_6_*)**

**Fig. S12. ^13^C NMR spectrum of compound 5 (100MHz, DMSO-*d*_6_)**

**Fig. S13.** **^1^H NMR spectrum of Compound 6 (400 MHz, DMSO-*d_6_*)**

**Fig. S14. ^13^C NMR spectrum of compound 6 (100MHz, DMSO-*d*_6_)**

 **Fig. S15. ^1^H NMR spectrum of Compound 7 (400 MHz, DMSO-*d_6_*)**

**Fig. S16. ^13^CNMR APT spectrum of compound 7 (100 MHz, DMSO *d_6_***
